# Research on Quality Inspection of PBF-LB 022Cr17Ni12Mo2 Steel Using Laser Ultrasonic Testing Technology

**DOI:** 10.3390/ma19173591

**Published:** 2026-08-24

**Authors:** Borui Zhang, Xianwei Yin, Chipeng Li, Chaochao Chen, Wanhong Li, Qiyuan Li, Anmin Yin

**Affiliations:** 1 Ningbo Iron and Steel Co., Ltd., Ningbo 315800, China; zbradang@163.com (B.Z.); yinxianwei@ningbosteel.com (X.Y.);; 2 Beijing University of Science and Technology Design and Research Institute Co., Ltd., Beijing 100083, China; ustbccc@163.com (C.C.); lwh125630@163.com (W.L.); 3 Institute of Engineering Technology, University of Science and Technology Beijing, Beijing 100083, China; 4 School of Mechanical Engineering and Intelligent Manufacturing, Ningbo University, Ningbo 315211, China; 17393518975@163.com

**Keywords:** laser ultrasonics, additive manufacturing, 022Cr17Ni12Mo2 steel, microstructure, mechanical properties

## Abstract

In this study, laser powder bed fusion (PBF-LB) 022Cr17Ni12Mo2 steel plates with dimensions of 50 mm × 50 mm × 2 mm were fabricated using a laser power of 206 W, a scanning speed of 900 mm/s, a hatch spacing of 90 μm, a layer thickness of 30 μm, and an interlayer scanning rotation of 67°. The specimens were then subjected to solution treatment at 900–1100 °C for 30 min and at 950 °C for 30–120 min. Unlike previous ultrasonic studies on additively manufactured metals, which mainly focused on defect detection, elastic-constant characterization, or residual stress evaluation, this work investigates whether solution-treatment-induced changes in grain size and dislocation density can be quantitatively reflected by laser-ultrasonic attenuation and further correlated with yield strength. Laser ultrasonic nondestructive testing using a 1064 nm pulsed laser with a pulse width of 8 ns and a pulse energy of 500 mJ was combined with metallographic observation, EBSD characterization, XRD analysis, tensile testing, and microhardness measurement. The results show that the solution-treated samples retained a single-phase γ-austenitic structure, while microstructural recovery, weakening of PBF-LB-induced cellular substructures, and partial annihilation of cell-wall dislocations led to a reduction in KAM-derived dislocation density from 2.04 × 10^14^ m^−2^ to 1.45 × 10^14^ m^−2^ and a decrease in yield strength from 466.9 MPa to 407.4 MPa. Within the present dataset, the EBSD-equivalent grain size showed an apparent positive correlation with ultrasonic attenuation, while the KAM-derived dislocation density showed an empirical negative correlation with ultrasonic attenuation. However, ultrasonic attenuation should be interpreted as a combined microstructure-sensitive response rather than as a response controlled only by EBSD-equivalent grain size or dislocation density. Based on the empirical correlations among ultrasonic attenuation, EBSD-equivalent grain size, KAM-derived dislocation density, and yield strength, a preliminary attenuation-based calibration model was established for the present solution-treated samples. The model should be regarded as an in-sample empirical calibration within the present experimental range rather than a general Hall–Petch-based predictive model. The model showed good in-sample fitting performance, with (R^2^) values higher than 0.85 and a maximum in-sample fitting error of 3.85%. However, because the model was established and assessed using the same eight solution-treatment conditions, it should be regarded as a preliminary calibration model within the present experimental range rather than a general predictive model. This study demonstrates the potential of laser ultrasonic attenuation for non-contact evaluation of microstructural and mechanical-property variations in solution-treated PBF-LB 022Cr17Ni12Mo2 steel.

## 1. Introduction

With the rapid development of additive manufacturing technology, laser powder bed fusion (PBF-LB), which is also referred to as selective laser melting (SLM) in some earlier studies and commercial systems, has been increasingly used to fabricate stainless steel components with complex geometries. In the present manuscript, the standard acronym PBF-LB is used throughout [1,2]. Compared with conventional manufacturing methods, SLM enables near-net-shape fabrication, high material utilization, a short production cycle, and the manufacture of complex structures [3]. However, the rapid melting and solidification during SLM inevitably introduce characteristic microstructural features, such as residual stress, pores, lack-of-fusion defects, anisotropic grains, melt-pool boundaries, cellular substructures, and high-density dislocations, which can significantly affect the mechanical performance of the fabricated components [4,5,6]. In this study, 022Cr17Ni12Mo2 steel, which is equivalent to 316L-type austenitic stainless steel, was selected as the research material [7]. As a typical low-carbon Cr–Ni–Mo austenitic stainless steel, 022Cr17Ni12Mo2 steel exhibits good corrosion resistance, ductility, weldability, and structural stability. Therefore, it is widely used in chemical equipment, marine engineering, petrochemical components, pressure vessels, heat exchangers, biomedical devices, and energy-related structures where corrosion resistance and mechanical reliability are required. With the development of SLM technology, this alloy can be further fabricated into complex or lightweight structural components. Nevertheless, its microstructure and mechanical properties are highly sensitive to SLM-induced features and subsequent solution treatment [8]. Therefore, rapid and nondestructive evaluation of solution-treated PBF-LB 022Cr17Ni12Mo2 steel is of practical significance for quality inspection and service-performance assessment.

The purpose of this study is to investigate whether solution-treatment-induced changes in grain size and dislocation density can be quantitatively reflected by laser-ultrasonic attenuation and further correlated with yield strength. It should be noted that PBF-LB components may contain internal self-balanced long-range residual stresses (LRRS), which can influence ultrasonic wave propagation and attenuation. In the present study, LRRS were not directly measured; therefore, the laser ultrasonic analysis is based on the working assumption that the relative attenuation variation among the solution-treated specimens is mainly associated with microstructural evolution rather than with LRRS alone. This assumption is considered reasonable because all specimens were fabricated with the same geometry, processing parameters, and 67° interlayer scanning rotation strategy, and were subsequently subjected to solution treatment. The scanning rotation strategy and heat treatment are expected to reduce the accumulation of macroscopic residual stresses compared with strongly directional scanning. Nevertheless, the possible contribution of LRRS to the measured attenuation cannot be completely separated from the effects of grain morphology, cellular substructures, dislocation density, and residual pores. This limitation is explicitly considered in the interpretation of the ultrasonic results. The central hypothesis is that ultrasonic attenuation can sensitively detect microstructural evolution and serve as a reliable indicator for predicting mechanical properties. Currently, the microstructure and mechanical properties of additively manufactured metallic materials are commonly characterized by metallographic observation, electron backscatter diffraction (EBSD), X-ray diffraction (XRD), tensile testing, and hardness testing. Although these methods provide accurate information, they generally require sample cutting, grinding, polishing, etching, or destructive testing, and are therefore unsuitable for rapid and in-line quality evaluation [9]. In contrast, nondestructive testing methods offer fast and noninvasive assessment of internal defects, microstructural evolution, and property variation, and have been increasingly applied to additively manufactured metals [10,11]. Among them, ultrasonic testing has attracted considerable attention due to its high sensitivity, strong penetration capability, wide material applicability, and operational safety [12,13]. Previous studies have demonstrated the potential of ultrasonic techniques in this field. Honarvar et al. [14] reviewed various ultrasonic testing methods for additively manufactured components. Zhang et al. [15] characterized the elastic constants of TC4 titanium alloy using laser ultrasound, and Zhan et al. [16] investigated heat-treatment-induced residual stress in laser-additively manufactured TC4. Nevertheless, most existing studies have focused on defect detection, elastic constants, or residual stress, while quantitative evaluation of mechanical properties based on microstructure-sensitive ultrasonic attenuation remains underexplored.

For PBF-LB 022Cr17Ni12Mo2 steel, solution treatment can modify grain size, grain-boundary distribution, cellular substructures, and dislocation density, thereby affecting both mechanical properties and ultrasonic scattering attenuation [17]. Based on this relationship, the scientific objective of this work is to establish a microstructure-informed laser-ultrasonic evaluation method for solution-treated PBF-LB 022Cr17Ni12Mo2 steel. To verify the hypothesis, scanning electron microscopy (SEM), EBSD, XRD, tensile testing, microhardness measurement, and laser ultrasonic testing were used in combination to reveal the relationships among microstructural evolution, mechanical properties, and ultrasonic attenuation. An enhanced ultrasonic signal-processing method was used to extract reliable attenuation features [18]. This study aims to provide a rapid, non-contact, and nondestructive quality inspection method for additively manufactured stainless steel components after post-processing heat treatment.

## 2. Materials and Experimental Methods

### 2.1. Experimental Materials and Sample Preparation

**Figure 1:**
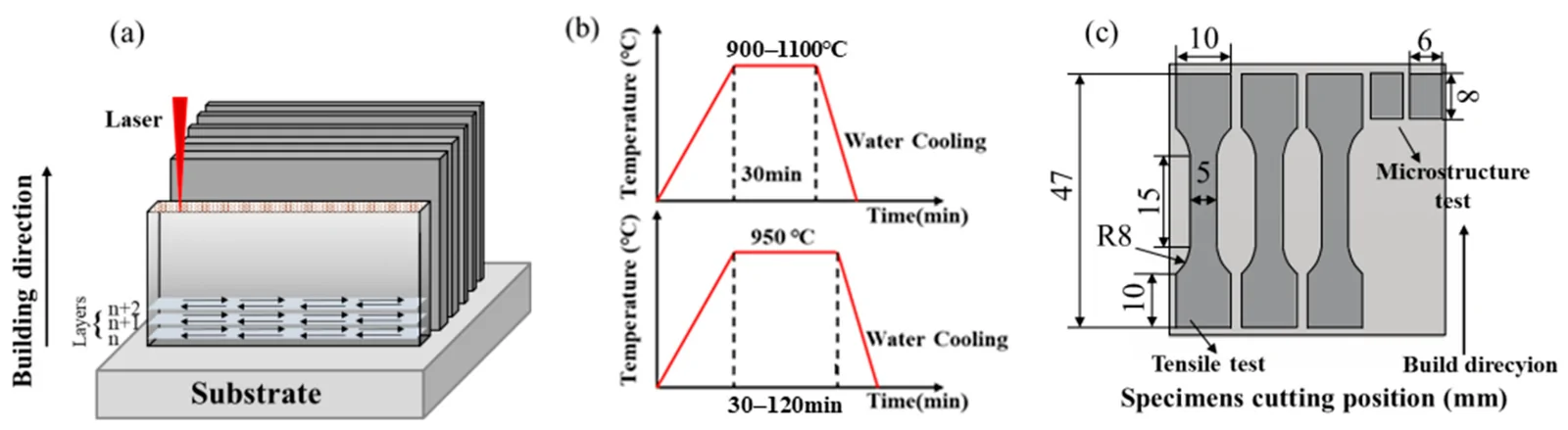
Experimental flow of PBF-LB 022Cr17Ni12Mo2 steel: (**a**) scanning paths, build direction, and substrate configuration; (**b**) solution treatment process; and (**c**) sample cutting layout and tensile specimen extraction position.

Gas-atomized 022Cr17Ni12Mo2 stainless steel powder with a particle size range of 10–45 μm was used as the feedstock for PBF-LB. The chemical composition of the powder is shown in Table 1. It meets the compositional requirements of low-carbon Cr–Ni–Mo austenitic stainless steel and is comparable to 316L-type stainless steel. Before fabrication, the powder was dried to reduce moisture-induced effects on powder spreading and melt-pool stability. The specimens were fabricated on a 022Cr17Ni12Mo2 stainless steel substrate with dimensions of 50 mm × 50 mm × 15 mm. Before PBF-LB fabrication, the substrate surface was mechanically cleaned and fixed on the build platform to ensure stable heat conduction and metallurgical bonding during deposition. The build direction was normal to the substrate surface, while the plate plane corresponded to the horizontal build plane. After fabrication, the deposited plates were separated from the substrate by wire electrical discharge machining. Plate-shaped specimens with dimensions of 50 mm × 50 mm × 2 mm were fabricated using an EOS M280 PBF-LB system under an argon protective atmosphere, as illustrated in Figure 1a. The main PBF-LB processing parameters were as follows: the laser power was 206 W, the scanning speed was 900 mm/s, the hatch spacing was 90 μm, the layer thickness was 30 μm, and the scanning direction was rotated by 67° between adjacent layers. These parameters were selected based on the recommended processing window for 316L-type stainless steel and the previous literature to obtain dense specimens with stable forming quality.

The present study mainly focuses on the relative changes in microstructure, mechanical properties, and laser-ultrasonic attenuation induced by solution treatment under consistent PBF-LB processing conditions. It should be noted that, due to the original experimental design and sample allocation, all as-fabricated specimens were subsequently subjected to solution treatment after laser ultrasonic testing, and no untreated as-fabricated specimen from the same batch was separately retained for SEM observation. Therefore, an as-received/as-fabricated micrograph corresponding to the same batch and the same preparation condition could not be provided in this revision. To avoid introducing unsupported microstructural evidence, a micrograph from a different batch or a non-identical condition was not used. The relative density of the as-fabricated PBF-LB deposits was measured to be 99.7%. This high relative density indicates that the amount of process-induced porosity was limited under the present processing conditions. Because laser ultrasonic attenuation is highly sensitive to pore-type defects, the density information is essential for interpreting the attenuation results. In the present study, the high relative density suggests that the measured attenuation variation is not mainly dominated by pores. Instead, it is discussed in relation to solution-treatment-induced microstructural evolution, including grain morphology, grain-boundary characteristics, cellular substructures, and dislocation density. Nevertheless, a minor contribution from residual pores cannot be completely excluded, and this limitation has been acknowledged in the discussion.

The as-fabricated specimens were subsequently subjected to solution treatment, as shown in Figure 1b and Table 2. The solution-treatment temperatures were 900, 950, 1000, 1050, and 1100 °C with a holding time of 30 min. In addition, 950 °C was selected to investigate the effect of different holding times of 30, 60, 90, and 120 min. All specimens were water-cooled after solution treatment. Subsequently, specimens for SEM, EBSD, XRD, tensile testing, and microhardness measurement were cut according to Figure 1c. EBSD characterization was performed with a step size of 0.8 μm. For each condition, three tensile specimens were tested, and ten hardness measurements were performed on each sample to obtain representative average values.

### 2.2. Laser Ultrasonic Testing

**Figure 2:**
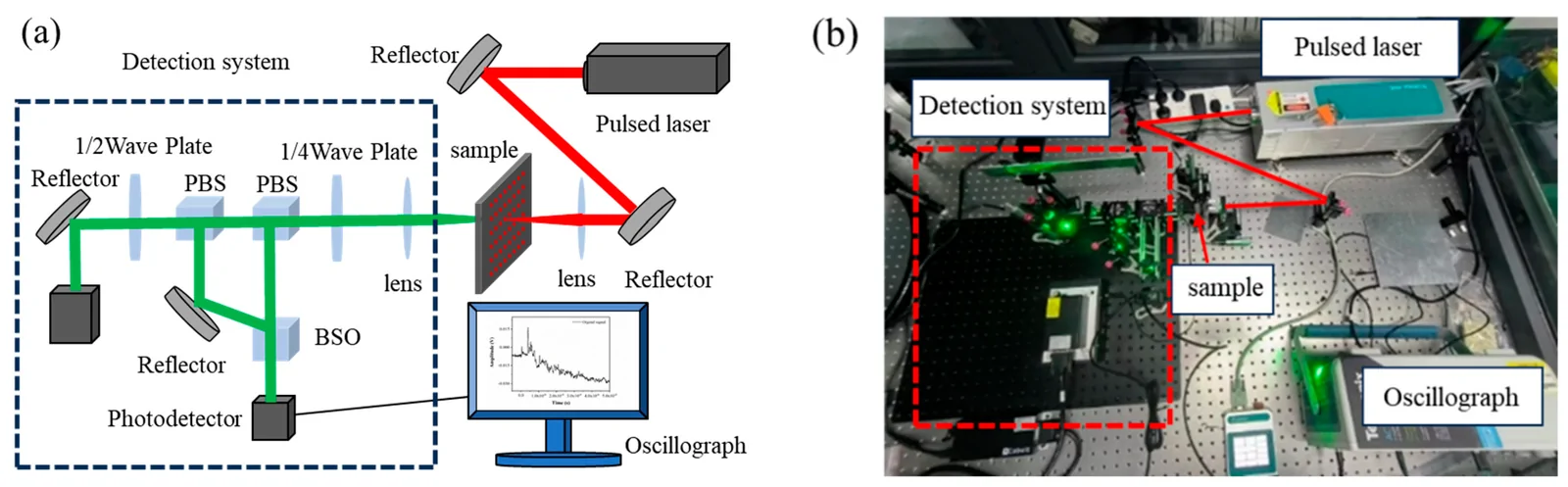
Laser ultrasonic testing system: (**a**) Detection principle; (**b**) experimental installation.

The laser ultrasonic detection system consisted of an ultrasonic optical excitation system, an optical detection system, and a signal acquisition system. A schematic of the laser ultrasonic detection system is shown in Figure 2. The ultrasonic excitation system utilized a Nd Q-switched pulsed laser with a wavelength of 1064 nm, a pulse width of 8 ns, and a pulse energy of 500 mJ [19]. These laser parameters were selected to generate detectable bulk longitudinal waves in the 2 mm-thick PBF-LB 022Cr17Ni12Mo2 steel plates while maintaining stable signal acquisition for attenuation analysis.

The ultrasonic detection system employed a two-wave mixing interferometer based on a BSO crystal and a 532 nm continuous laser. This non-contact optical detection configuration was used because it avoids couplant-induced variability and is suitable for detecting weak ultrasonic signals from additively manufactured metallic specimens. The laser beam emitted from the pulsed laser was reflected by a mirror and then focused onto the surface of the PBF-LB 022Cr17Ni12Mo2 steel sample using a convex lens to generate ultrasonic waves. The generated ultrasonic waves propagated through the thickness direction of the sample, and the detection system positioned on the opposite side received the transmitted longitudinal-wave signals in a non-contact manner. The through-transmission configuration was adopted because it provides relatively stable bulk-wave signals and multiple echoes, which are suitable for calculating ultrasonic attenuation coefficients and evaluating solution-treatment-induced microstructural changes.

### 2.3. Microstructural Characterization and Mechanical Property Testing

Laser ultrasonic testing was performed on the intact plate specimens before sectioning. After ultrasonic testing, the specimens for SEM, EBSD, XRD, tensile testing, and microhardness measurement were sectioned from representative regions of the plates according to Figure 1c. The sectioning was carried out using wire electrical discharge machining to reduce additional mechanical deformation and thermal damage. It should be noted that sectioning and mechanical preparation may partially relax the original self-balanced long-range residual stresses (LRRS) in the PBF-LB deposits. Therefore, the microhardness and microstructural characterization results obtained from sectioned samples are used as reference information for the solution-treated material state, whereas laser ultrasonic attenuation was measured on the intact plates before destructive sample preparation. For metallography, samples were embedded, ground with 2500-grit sandpaper, polished, and etched with aqua regia (HCl:HNO_3_ = 3:1) for 30 s to reveal melt-pool morphology and grain-boundary features. Microstructure was observed using a Hitachi SU5000 SEM. For EBSD characterization, the specimens were further mechanically polished and then electrolytically polished using a solution of glycerol, perchloric acid, and ethanol with a volume ratio of 1:2:7 at 15 V and 1.5 A for 30 s to remove the surface deformation layer. EBSD mapping was performed with a step size of 0.8 μm, and the obtained data were analyzed using Aztec Crystal software, version 2.1. Grain boundaries with misorientation angles of 2–15° were defined as low-angle grain boundaries, whereas those with misorientation angles higher than 15° were defined as high-angle grain boundaries. Grain size, grain-boundary fraction, inverse pole figure maps, texture intensity, and kernel average misorientation maps were extracted to evaluate the effects of solution treatment on grain morphology, boundary distribution, local misorientation, and dislocation-density evolution.

**Figure 3:**
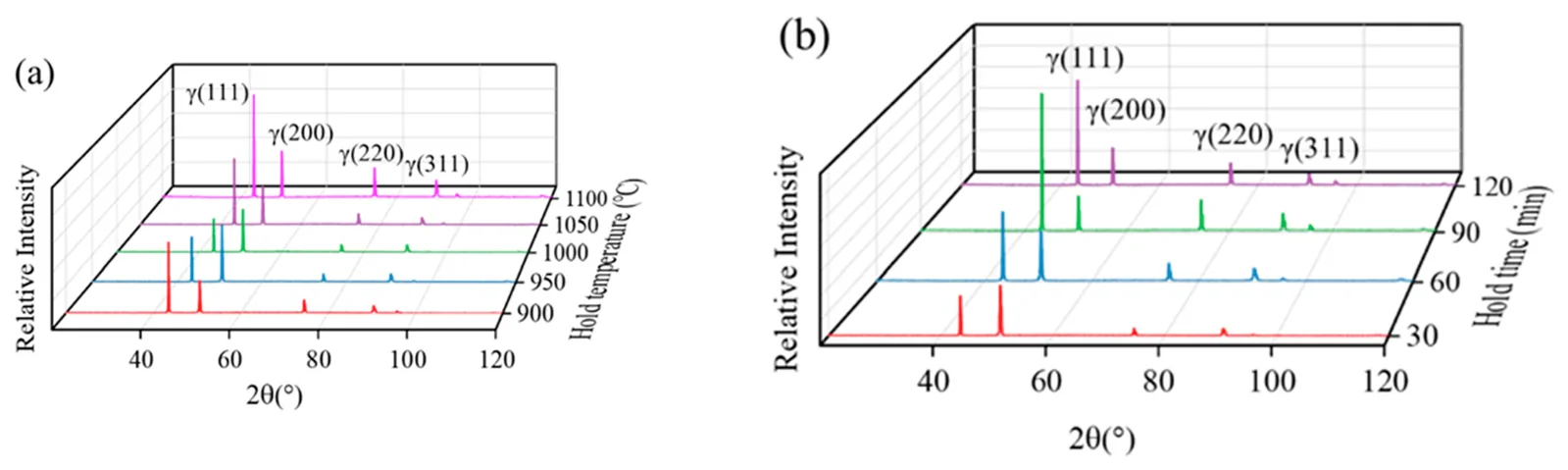
Phase structure analysis of PBF-LB 022Cr17Ni12Mo2 steel after solution treatment: (**a**) different holding temperatures and (**b**) different holding times.

Considering the original dimensions of the PBF-LB-fabricated plates (50 mm × 50 mm × 2 mm), small-sized flat tensile specimens were designed and machined with reference to GB/T 228.1-2021, Metallic materials—Tensile testing—Part 1: Method of test at room temperature. As shown in Figure 1c, the tensile specimens were extracted from the central region of the fabricated plates in the horizontal build plane. The longitudinal direction of the gauge section was parallel to the build plane and perpendicular to the build direction. Along the build direction, each tensile specimen corresponded to the full plate thickness of 2 mm. The total length of the tensile specimen was 47 mm, the grip-end width was 10 mm, the gauge length was 15 mm, the gauge width was 5 mm, and the specimen thickness was 2 mm. Three tensile specimens were tested for each solution-treatment condition, and the average values were calculated to reduce the influence of local microstructural heterogeneity. Mechanical properties were evaluated using an MTS810 universal testing machine, with three tensile specimens tested for each solution-treatment condition. The average values were calculated to reduce the influence of local microstructural heterogeneity and improve statistical reliability. Mechanical properties were evaluated using an MTS810 universal testing machine with three tensile specimens per condition, and hardness was measured with an HXD-1000TMC/LCD microhardness tester under a 100 g load for 15 s with ten indentations per sample. Average values were calculated to reduce the influence of local microstructural heterogeneity and improve statistical reliability. The phase structure was analyzed using a D8 Advance X-ray diffractometer with Cu Kα radiation, and the XRD results were identified using MDI Jade 6 software. XRD analysis was performed on the surface of the PBF-LB 022Cr17Ni12Mo2 steel along the build direction, and the results are shown in Figure 3.

**Figure 4:**
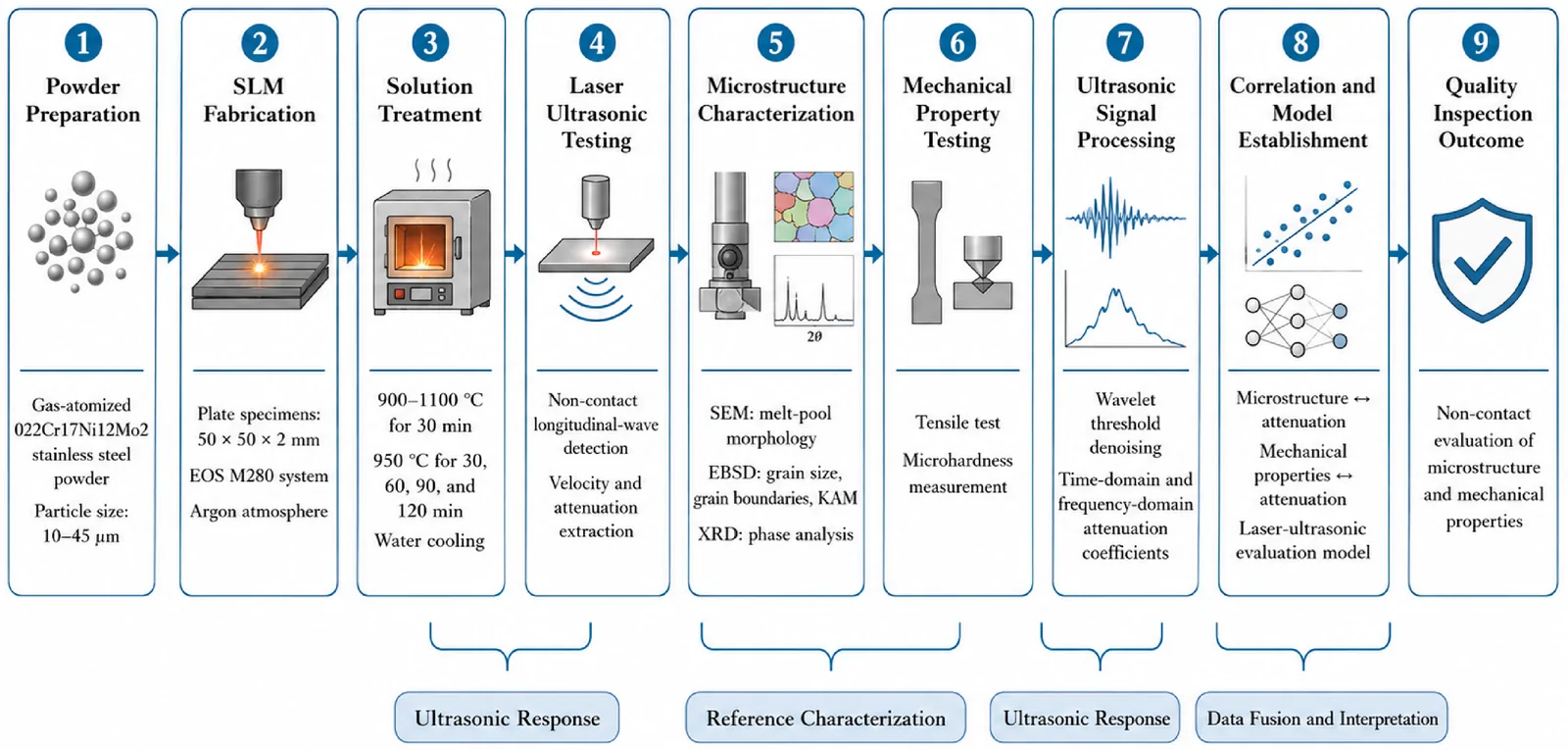
Workflow diagram of the experimental procedure and laser-ultrasonic evaluation method for PBF-LB 022Cr17Ni12Mo2 steel.

It can be seen that the samples treated at different solution temperatures and holding times mainly exhibit a γ-austenite phase, with the dominant diffraction peaks corresponding to the γ (111) and γ (200) planes. With increasing solution-treatment temperature and holding time, no new diffraction peaks are detected, indicating that no obvious new phase or secondary precipitate is formed under the solution-treatment conditions used in this study. Only the diffraction peak intensity changes to some extent. Therefore, the solution-treated PBF-LB 022Cr17Ni12Mo2 steel remains a single-phase austenitic structure, and the subsequent variations in mechanical properties and ultrasonic response are mainly related to microstructural recovery, weakening of cellular substructures, and reduction in dislocation density rather than phase transformation or precipitate dissolution. The experimental procedure is shown in Figure 4.

## 3. Results and Discussion

### 3.1. Microstructural Recovery and Dislocation Evolution After Solution Treatment

#### 3.1.1. Microstructure and Phase Structure

**Figure 5:**
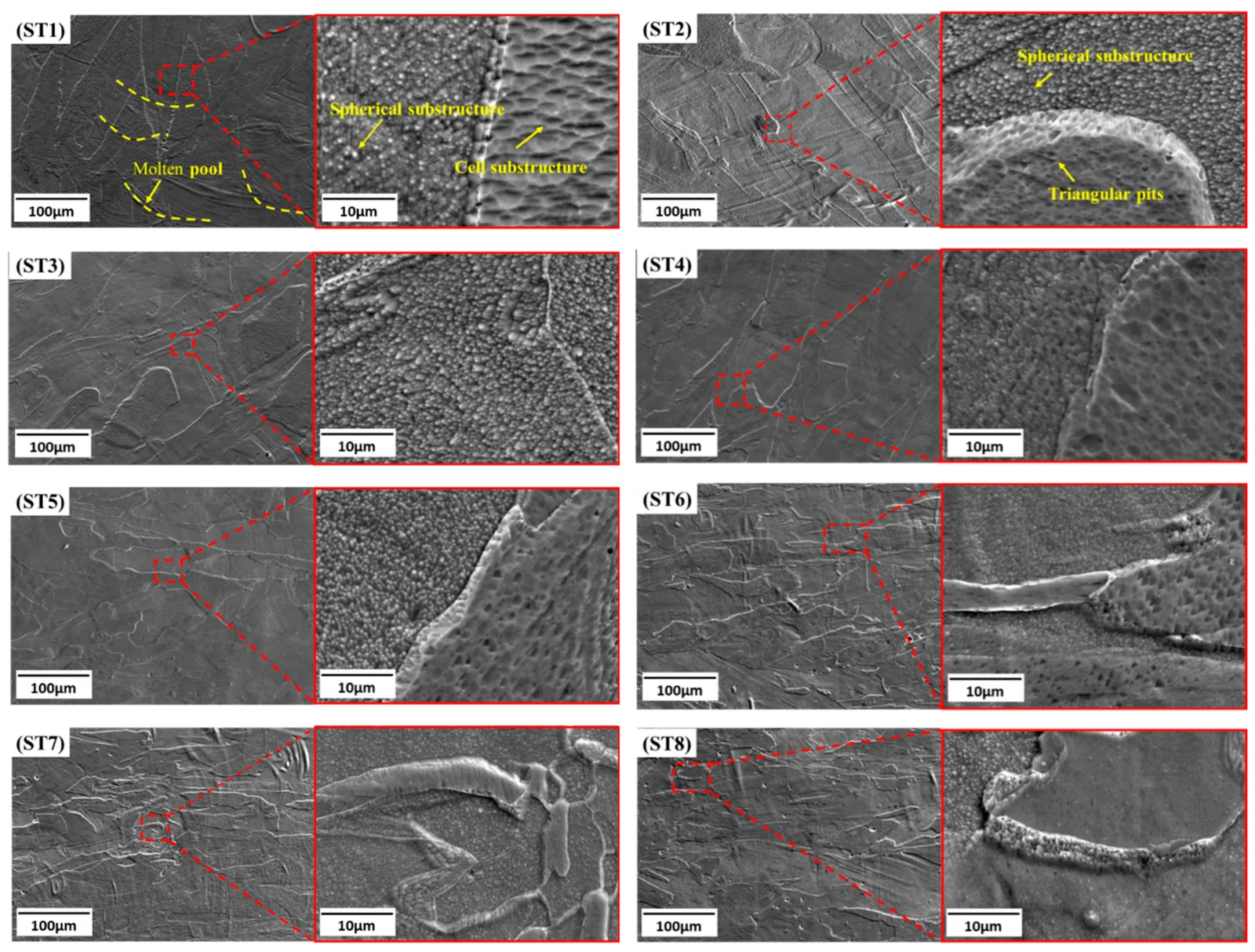
Microstructure evolution of PBF-LB 022Cr17Ni12Mo2 steel after solution treatment.

The microstructure of PBF-LB 022Cr17Ni12Mo2 steel parallel to the build direction was observed by SEM. The microstructural evolution after solution treatment is shown in Figure 5. After solution treatment, the microstructure of PBF-LB 022Cr17Ni12Mo2 steel is irregular strip-like, and the fish-scale-like molten pool morphology gradually disappears. During the solution treatment at 900 °C (ST1), there was a small amount of fish-scale-like molten pool morphology on the surface of the microstructure, and cellular subcrystalline structures were present inside the grains. The grain boundaries were spherical structures and triangular point-like pit structures. As the solution treatment temperature rose to 950 °C (ST2), the fish-scale-like molten pool morphology completely disappeared and gradually transformed into an irregular columnar recrystallized structure. The microstructure at the grain boundaries did not change significantly and still remained as spherical and triangular point-like pit structures. And as the temperature continues to rise, at 1000 °C (ST3), 1050 °C (ST4), and 1100 °C (ST5), the recrystallized structure gradually grows, the grain size increases, and the uniformity of the microstructure increases.

After solution treatment at 950 °C for 30 min (ST2), 60 min (ST6), 90 min (ST7), and 120 min (ST8), the microstructure of PBF-LB 022Cr17Ni12Mo2 steel showed limited variation with increasing holding time. This suggests that the original melt-pool morphology and cellular substructures were progressively weakened during solution treatment. The term “dissolution” is avoided here because no precipitate dissolution was identified by XRD. Instead, the observed microstructural change is more appropriately attributed to microstructural recovery, partial disappearance of cell-wall contrast, and annihilation or rearrangement of dislocations.

#### 3.1.2. Crystal Orientation and Grain Size

**Figure 6:**
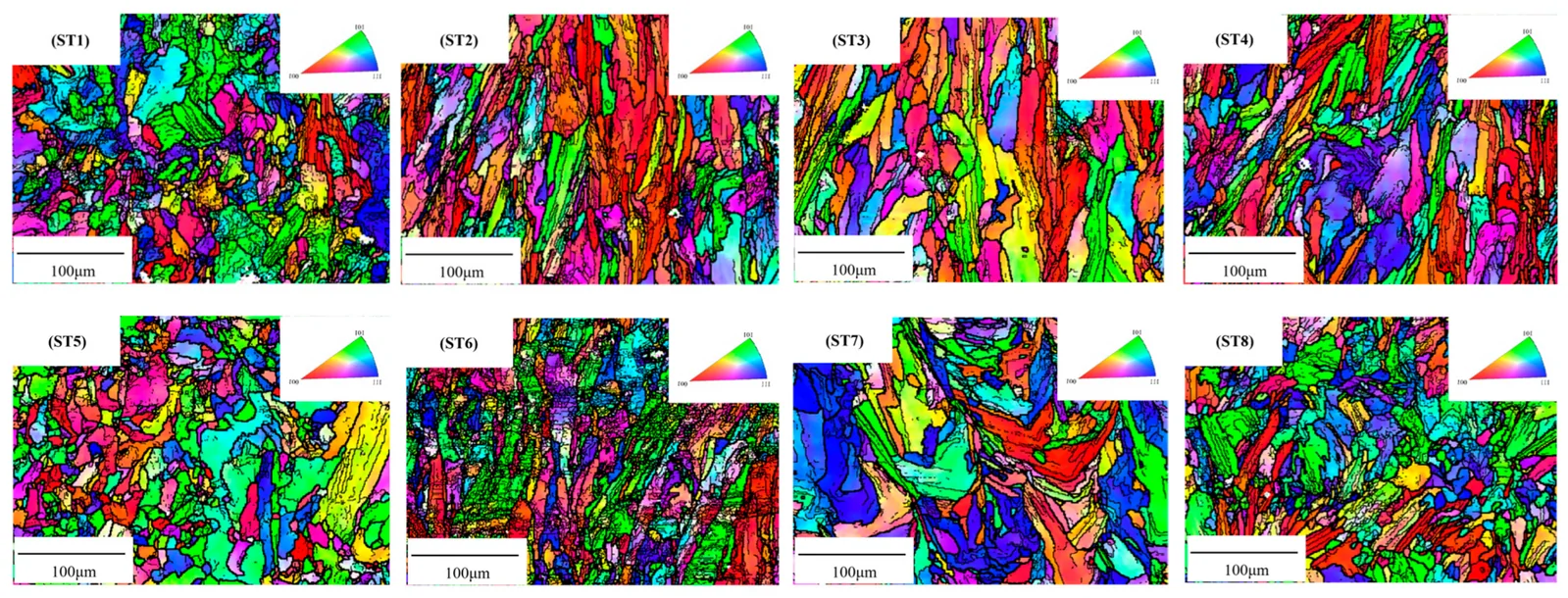
Crystal orientation analysis of PBF-LB 022Cr17Ni12Mo2 steel after solution treatment.

EBSD data of the solution-treated PBF-LB 022Cr17Ni12Mo2 steel were analyzed using Aztec Crystal software, as shown in Figure 6. From the figure, the grains of the PBF-LB 022Cr17Ni12Mo2 steel after solution treatment transition from irregular, elongated strips to equiaxed crystals, with no distinct pattern in crystal orientation, can be observed. The statistical results of the average grain size are presented in Table 3. As shown in Table 3, the grain size varies with the solution-treatment conditions rather than showing a strictly monotonic increase under all conditions. For the samples treated at different temperatures for 30 min, the average grain size changes from 12.82 μm in ST1 to 16.31 μm in ST5, with a maximum value of 19.29 μm in ST3. For the samples treated at 950 °C for different holding times, the average grain size changes from 14.51 μm in ST2 to 16.22 μm in ST8. These results indicate that solution treatment affects grain morphology and recrystallization behavior, but the grain-size evolution is also influenced by local microstructural heterogeneity in the PBF-LB specimens.

#### 3.1.3. Grain Boundary and Dislocation Density

**Figure 7:**
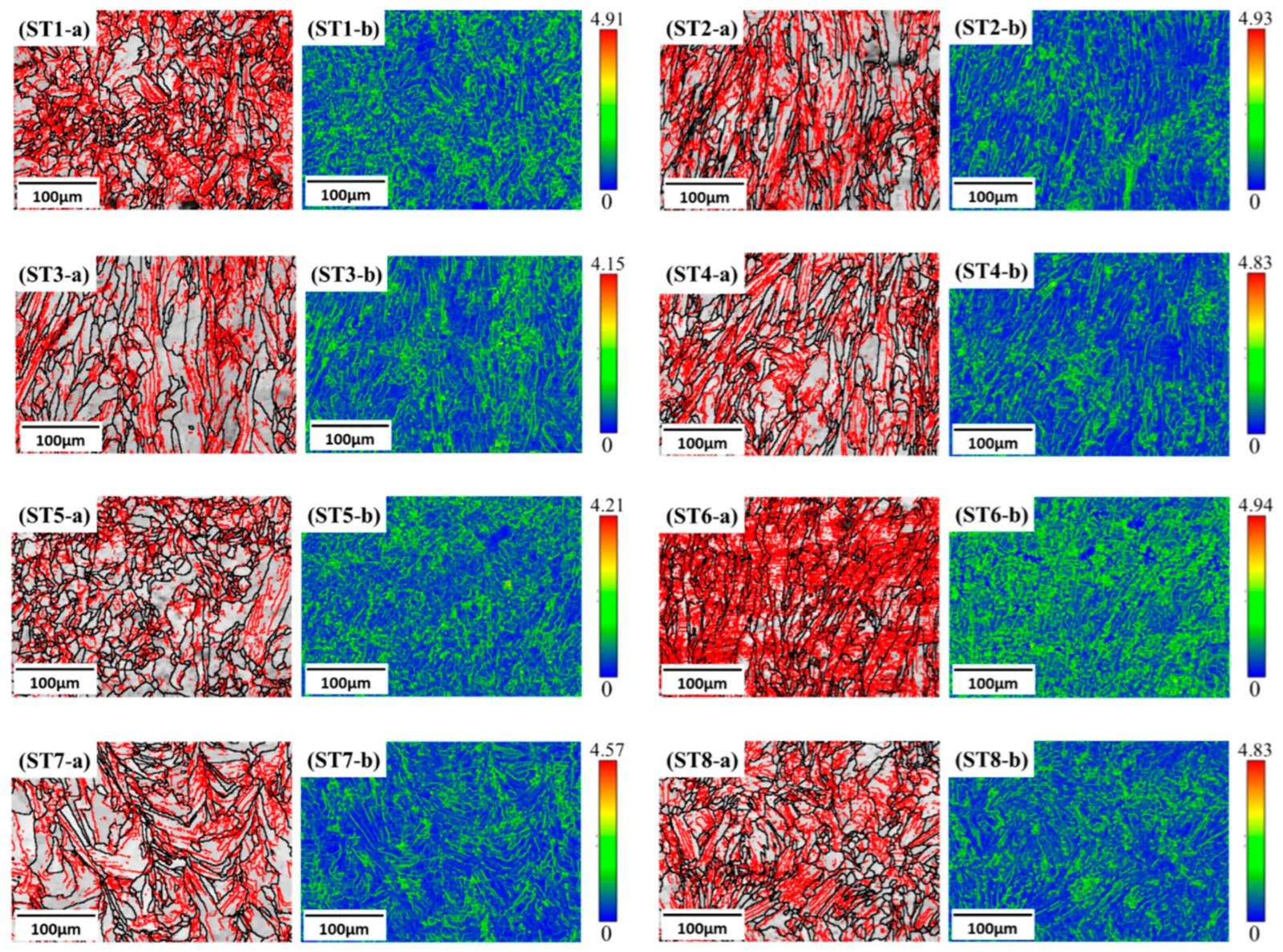
The grain boundary distribution and dislocation density distribution of PBF-LB 022Cr17Ni12Mo2 steel after solution treatment: (**a**) grain boundary distribution; (**b**) kernel average microstructure.

The boundary distribution map and kernel average misorientation (KAM) map of the PBF-LB 022Cr17Ni12Mo2 steel samples subjected to different solution treatments were obtained through Aztec Crystal software analysis, as shown in Figure 7. In these maps, the red lines represent low-angle grain boundaries (2–15°), while the black lines correspond to high-angle grain boundaries (>15°). PBF-LB 022Cr17Ni12Mo2 steel typically exhibits a cell-shaped dislocation entanglement, with low-angle grain boundaries representing a collection of dislocations at the grain boundaries. A higher density of low-angle grain boundaries corresponds to an increased dislocation density, greater stored deformation energy, and consequently higher strength and hardness of the material, but lower plastic toughness [20]. The dislocation density can be calculated using Formula (1) [21,22]. The statistical results for low-angle grain boundaries and the corresponding dislocation density calculations after different solution treatments are presented in Table 3.

(1) $$\rho =\frac{2\theta }{ub}$$

where *ρ* is the dislocation density, *θ* is the average misorientation from the KAM map and should be expressed in radians, *u* is the EBSD scanning step length, and *b* is the Burgers vector (0.256 nm).

From Figure 7, it can be clearly found that the dislocation density is also aggregated in the area where the low-angle grain boundaries are aggregated. As shown in Table 3, for the samples solution-treated at 900–1100 °C for 30 min, the fraction of low-angle grain boundaries decreased from 52.0% in ST1 to 35.9% in ST5, while the KAM-derived dislocation density decreased from 2.04 × 10^14^ m^−2^ to 1.45 × 10^14^ m^−2^. Although local fluctuations exist among different solution-treatment conditions, the overall reduction in low-angle grain-boundary fraction and KAM-derived dislocation density indicates the occurrence of recovery, dislocation rearrangement, and partial annihilation of cell-wall dislocations during solution treatment. For the samples treated at 950 °C for different holding times, the low-angle grain-boundary fraction and dislocation density also vary with holding time, reflecting the combined effects of microstructural recovery, recrystallization, and local heterogeneity. The cellular substructures were weakened rather than interpreted as being dissolved in the sense of precipitate dissolution. This interpretation is consistent with the decrease in yield strength and hardness and the improvement in ductility after solution treatment. For the samples treated at 950 °C for different holding times, the low-angle grain-boundary fraction and dislocation density also vary with holding time, reflecting the combined effects of microstructural recovery, recrystallization, and local heterogeneity. From the KAM map, it is also evident that the proportion of low-angle grain boundaries decreases, and the dislocation density at the grain boundaries diminishes. This is attributed to the gradual growth of grains with increasing temperature. As the temperature rises, many fine grains coalesce into larger grains, causing the dissolution of the cellular entangled sub-grain structure, leading to a reduction in dislocation density.

### 3.2. Effect of Microstructure Evolution on Mechanical Properties

**Figure 8:**
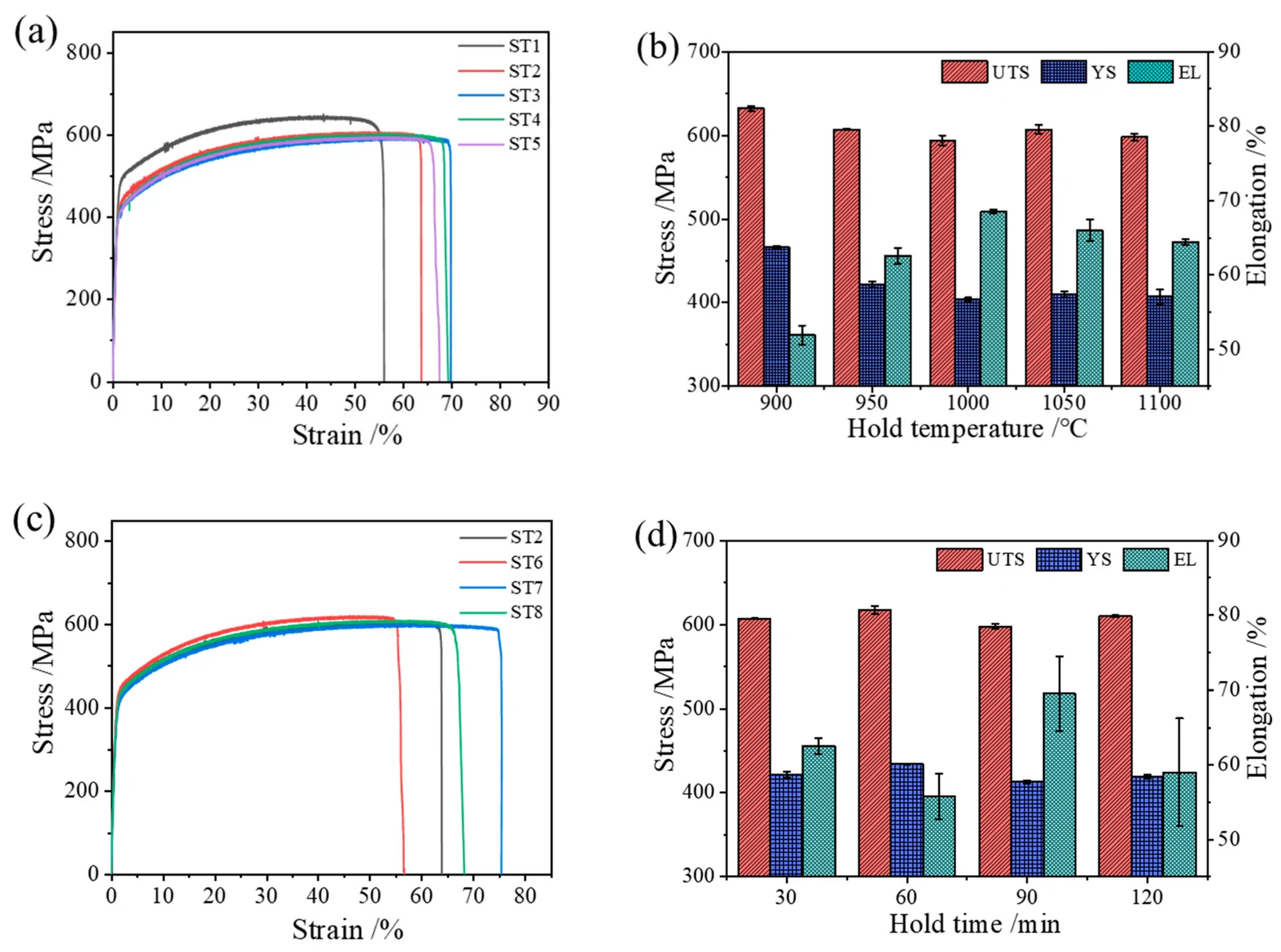
Mechanical properties of PBF-LB 022Cr17Ni12Mo2 steel after solution treatment: (**a**,**c**) stress–strain curves under different holding temperatures and holding times; (**b**,**d**) mechanical-property variations under different holding temperatures and holding times.

The mechanical properties of PBF-LB 022Cr17Ni12Mo2 steel samples after different solution treatments were evaluated through tensile and hardness tests, and the results are shown in Figure 8. The corresponding mechanical property parameters are presented in Table 4. The tensile properties of the PBF-LB 022Cr17Ni12Mo2 steel samples subjected to solution treatment at temperatures ranging from 900 °C to 1100 °C are shown in Figure 8b. As observed in the figure, with increasing holding temperature, the yield strength (YS) decreases from 466.9 MPa to 407.46 MPa, and the ultimate tensile strength (UTS) decreases from 632.3 MPa to 597.9 MPa, while the elongation (EL) increases from 51.9% to 64.4%. It is well known that the mechanical properties of a material are primarily determined by its microstructure. Grain size and dislocation density are considered two crucial factors affecting the mechanical properties of PBF-LB 022Cr17Ni12Mo2 steel [23].

According to the Hall–Petch relationship, the strengthening effect of grain boundaries on mechanical properties is inversely proportional to the grain size, meaning that as the grain size increases, the strength decreases. As the temperature increases, the highly concentrated dislocations in the material are reduced, which in turn lowers the resistance to dislocation slip. The decrease in dislocation density also contributes to the reduction in the material’s strength. The hardness of the material is closely related to dislocation density: the higher the dislocation density, the greater the hardness. A decrease in the content of low-angle grain boundaries leads to a reduction in dislocation density, which in turn results in a decrease in hardness from 246.3 HV to 223.8 HV. The highest hardness value of ST1, 246.3 ± 5.6 HV, is mainly attributed to the relatively low solution-treatment temperature. At 900 °C, part of the PBF-LB-induced cellular substructures, low-angle grain boundaries, and high-density dislocations were still retained, which increased the resistance to dislocation motion and enhanced dislocation strengthening. As the solution-treatment temperature increased, dislocation recovery and partial annihilation of cell-wall dislocations occurred, and the cellular-substructure contrast was weakened. Consequently, the dislocation density and hardness decreased.

The tensile properties of the PBF-LB 022Cr17Ni12Mo2 steel samples subjected to solution treatment at 950 °C for different holding times are shown in Figure 8d. For the samples solution-treated at 950 °C for different holding times, the mechanical properties fluctuate with holding time rather than changing monotonically. The yield strength changes from 421.7 MPa in ST2 to 434.1 MPa in ST6, then decreases to 413.1 MPa in ST7, and slightly increases to 419.5 MPa in ST8. When the holding time increased from 30 min to 60 min, the tensile strength rose from 607 MPa to 617.2 MPa, the hardness increased from 232.3 HV to 234.8 HV, and the elongation decreased from 62.5% to 55.7%. At a holding time of 90 min, as the grain size increased, the proportion of small-angle grain boundaries decreased, and the high-density dislocations at the grain boundaries diminished. Consequently, the resistance to dislocation slip at the grain boundaries decreased. As a result, the tensile strength decreased to 597.7 MPa, the hardness dropped to 226.1 HV, and the elongation increased to 69.5%.

The above mechanical-property variation is consistent with previous studies on heat-treated PBF-LB 316L stainless steels. It has been reported that post-build heat treatment promotes recovery, recrystallization, weakening of cellular substructures, and partial annihilation or rearrangement of cell-wall dislocations in PBF-LB 316L stainless steel, thereby reducing dislocation strengthening and improving ductility [24,25]. As a result, the yield strength and hardness generally decrease, while the ductility tends to improve. In the present study, the decrease in yield strength from 466.9 MPa to 407.4 MPa and the decrease in hardness from 246.3 HV to 223.8 HV with increasing solution-treatment temperature are therefore in good agreement with the microstructure–property relationship reported for heat-treated PBF-LB 316L-type stainless steels. This comparison further supports the conclusion that the mechanical-property evolution of PBF-LB 022Cr17Ni12Mo2 steel is mainly governed by grain growth, reduction in low-angle grain boundaries, and decrease in dislocation density.

It should also be noted that the microhardness measurements were performed on sectioned and mechanically prepared samples, whereas laser ultrasonic testing was conducted on the intact plates before sectioning. Cross-section cutting and surface preparation may partially relax LRRS, and may therefore influence the local hardness response. For this reason, the hardness results are used as supporting evidence for the relative microstructural evolution rather than as a direct one-to-one counterpart of the ultrasonic attenuation measured on the intact deposits. The comparison between hardness and ultrasonic attenuation should therefore be understood from their common sensitivity to microstructural recovery, dislocation-density reduction, and cellular-substructure evolution.

### 3.3. Effect of Microstructure Evolution on Ultrasonic Signals

**Figure 9:**
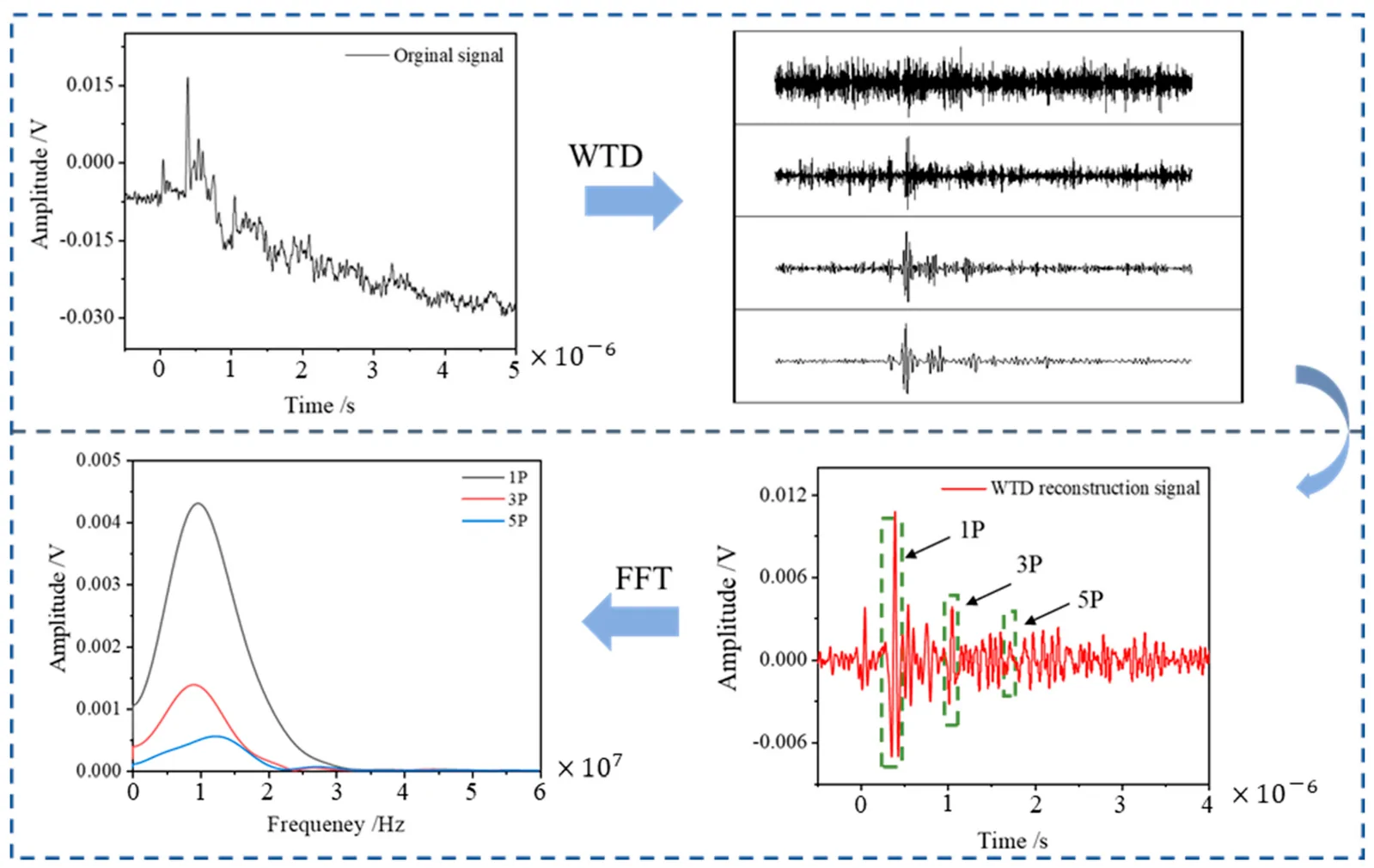
Wavelet-threshold denoising processing of laser-ultrasonic longitudinal-wave signals.

The ultrasonic characteristic parameters obtained through laser ultrasonic testing are closely related to the microstructure of the sample. Changes in the microstructure have a significant impact on these ultrasonic characteristic parameters [26]. In this paper, the wavelet threshold denoising (WTD) method is used to denoise the laser ultrasonic longitudinal wave signal. The db5 wavelet function is selected as the basis function, and the signal is decomposed into five layers. A smoothing function is then applied to obtain the denoised signal. The original signals of PBF-LB 022Cr17Ni12Mo2 steel after different solution treatments, together with the wavelet-denoised signals, are shown in Figure 9. The time difference between the first echo (1P) and the second echo (3P) is selected and substituted into the ultrasonic velocity calculation Formula (2) to determine the ultrasonic velocity between successive echoes.

(2) $$v=\frac{2L}{t_{i}-t_{i-1}}$$

where *L* is the sample thickness and $t_{i}$ is the time of the *i* echo signal.

The wave peaks of the first echo (1P), the second echo (3P), and the third echo (5P) are selected and substituted into the ultrasonic attenuation calculation Formula (3) to calculate the ultrasonic time-domain attenuation coefficient. Then, the three echoes are processed using the fast Fourier transform (FFT) function to obtain the spectrogram. The peaks of the three echoes are selected again and substituted into the ultrasonic attenuation calculation Formula (3) to derive two sets of ultrasonic frequency-domain attenuation coefficients. The results are shown in Table 5.

(3) $$\alpha =\frac{20}{2L}\log_{10}(\frac{A_{i}}{A_{i+1}})$$

where $A_{i}$ and $A_{i+1}$ are the amplitudes of two successive echoes, 2L is the additional propagation distance between adjacent echoes, and *α* is the ultrasonic attenuation coefficient. In this study, the attenuation coefficient α was calculated from the amplitude decrease between two successive longitudinal-wave echoes. Therefore, a larger α indicates a stronger amplitude loss per unit propagation distance and a higher ultrasonic attenuation level.

As shown in Table 5, the attenuation coefficients calculated from successive longitudinal-wave echoes generally vary with the solution-treatment condition. This indicates that the amplitude loss during ultrasonic propagation is sensitive to the microstructural state of PBF-LB 022Cr17Ni12Mo2 steel. However, the attenuation coefficient should not be interpreted as being controlled by EBSD-equivalent grain size alone. In PBF-LB austenitic stainless steel, the ultrasonic response may be affected simultaneously by residual pores, elongated grains, grain-boundary morphology, cellular substructures, cell-wall dislocation networks, local dislocation density, texture, and possible LRRS. Therefore, the measured attenuation represents a combined microstructure-sensitive response. Within the present dataset, EBSD-equivalent grain size is used as an apparent statistical descriptor, while cellular substructures and dislocation-related recovery are also considered in the interpretation.

It should be emphasized that the as-fabricated deposits exhibited a high relative density of 99.7%. Therefore, although ultrasonic attenuation is sensitive to pores, the influence of porosity is considered limited in the present specimens. Therefore, the attenuation variation after solution treatment is discussed mainly as a microstructure-sensitive response related to grain morphology, grain-boundary distribution, cellular substructures, and dislocation-density evolution. However, the possible contribution of residual pores is not completely excluded.

In addition, the possible influence of LRRS should be considered. The laser ultrasonic attenuation measured in this work may contain contributions from residual stress, microstructural scattering, dislocation-related damping, and residual porosity. Since LRRS were not independently quantified, the attenuation coefficient is not interpreted as a direct measure of dislocation density or grain size alone. Instead, it is used as an empirical ultrasonic descriptor reflecting the combined effects of the microstructural state after solution treatment.

### 3.4. Laser Ultrasonic Testing of Microstructure and Mechanical Properties

#### 3.4.1. Laser Ultrasonic Testing Model of Microstructure

Ultrasonic characteristic parameters contain valuable microstructure information of the samples, which can be used to infer material properties such as grain size, morphology, and dislocation density. During the propagation of ultrasonic waves within the material, phenomena such as reflection, scattering at microstructural interfaces, and absorption of ultrasonic energy by the material lead to a gradual weakening of the ultrasonic wave energy. This attenuation occurs according to a specific pattern. The attenuation of ultrasonic waves due to grain size is primarily attributed to scattering. The relationship between grain size and ultrasonic attenuation is given by Equation (4) [27]:

(4) $$\alpha =\left\{\begin{array}{l} K_{r}D^{3}f^{4},D\ll \lambda \\ K_{s}Df^{2},D\approx \lambda \\ K_{d}/D,D\gg \lambda \end{array}\right.$$

where *α* is the ultrasonic attenuation coefficient; $K_{r}$, $K_{s}$, $K_{d}$ are constants related to the material; f is the ultrasonic frequency; *D* is the average grain size; and *λ* is the wavelength of the ultrasonic wave.

In this experimental system, the highest frequency of the ultrasonic wave is approximately 30 MHz, and the velocity of the longitudinal wave is about 5670 m/s. Using Formula (5), the wavelength of the ultrasonic wave can be calculated to be 189 μm [28].

(5) λ=v/f

where *λ* is the wavelength of the ultrasonic wave and *v* is the ultrasonic longitudinal wave velocity.

**Figure 10:**
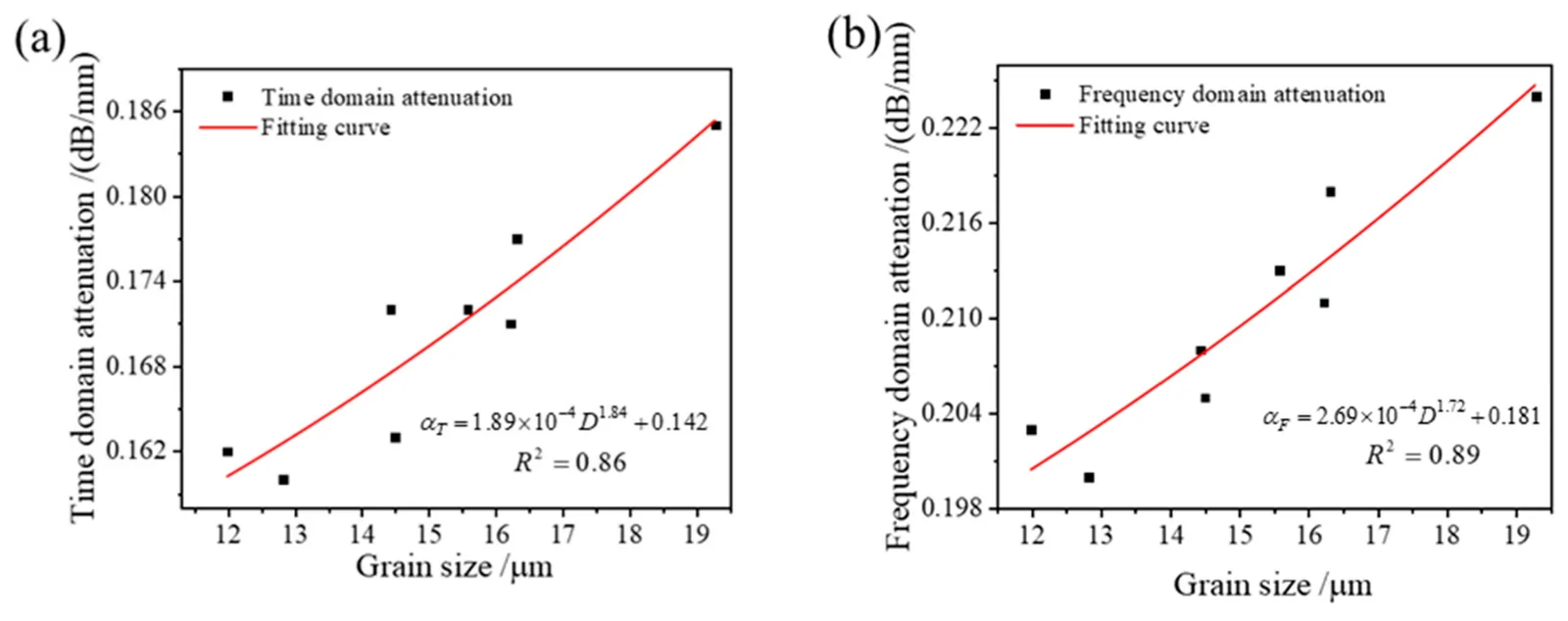
Correlation analysis between the ultrasonic attenuation coefficient and grain size: (**a**) time domain attenuation; (**b**) frequency domain attenuation.

The average grain size of PBF-LB 022Cr17Ni12Mo2 steel after different solution treatments ranges from approximately 10 μm to 20 μm, which is significantly smaller than the ultrasonic wavelength of 189 μm. Based on the relationship between ultrasonic attenuation and average grain size, when the grain size is much smaller than the ultrasonic wavelength, the ultrasonic scattering mechanism follows Rayleigh scattering. From the previous microstructure observations, it can be seen that the microstructure after solution treatment remains uneven, with some large grains ranging from 150 μm to 200 μm. When the ultrasonic wave propagates through these larger grains, random scattering occurs. Therefore, the scattering mechanism affecting the ultrasonic attenuation coefficient and grain size in this study is a combination of Rayleigh scattering and random scattering. The ultrasonic attenuation evaluation model for grain size, based on different scattering mechanisms, is given in Equation (6) [29]. To validate the feasibility of the evaluation model, a fitting analysis is conducted using the attenuation coefficients and average grain sizes of the first and second echoes. The results are presented in Figure 10.

(6) $$\alpha =aD^{n}+\alpha_{0}$$

where *α* is the ultrasonic attenuation coefficient, *a* is a constant related to the material, *n* is different scattering mechanisms (−1~3), *D* is the average grain size, and $\alpha_{0}$ is absorption attenuation.

It can be observed from Figure 10 that the ultrasonic time-domain and frequency-domain attenuation coefficients show an apparent positive correlation with the EBSD-equivalent grain size within the present solution-treated sample set. This relationship should be regarded as an empirical correlation rather than as direct evidence that the large, irregular EBSD grains are the only dominant scattering units. In PBF-LB 316L-type stainless steel, grains usually exhibit elongated and irregular morphologies and are internally filled with cellular substructures. The cell walls and their associated dislocation density may also contribute significantly to ultrasonic scattering and attenuation. Therefore, the EBSD-equivalent grain size is used here as a convenient statistical descriptor of microstructural evolution, while the physical origin of attenuation is attributed to the combined effects of grain-boundary scattering, cellular-substructure weakening, dislocation recovery, residual porosity, texture, and possible LRRS.

The fitting formulas for the ultrasonic time-domain attenuation coefficient and frequency-domain attenuation coefficient as a function of average grain size are obtained as follows:

(7) $$\alpha_{T}=1.89\times 10^{-4}D^{1.84}+0.142$$

(8) $$\alpha_{F}=2.69\times 10^{-4}D^{1.72}+0.181$$

where $\alpha_{T}$ is the time domain attenuation coefficient and $\alpha_{F}$ is the frequency domain attenuation coefficient.

It can be observed from Figure 10 that both the ultrasonic time-domain attenuation and frequency-domain attenuation are positively correlated with grain size. As the grain size increases, the attenuation coefficient also increases. The goodness of fit between grain size and both ultrasonic time-domain and frequency-domain attenuation is above 0.86. The positive correlation between grain size and ultrasonic attenuation is also consistent with previous ultrasonic studies on 316L stainless steel and other polycrystalline metallic materials. According to ultrasonic scattering theory, grain boundaries act as scattering centers during ultrasonic propagation, and the attenuation coefficient is strongly affected by the relationship between ultrasonic wavelength and grain size. Wang et al. [30] reported that the Rayleigh-wave attenuation coefficient increased with increasing grain size in heat-treated 316L stainless steel, indicating that ultrasonic attenuation is sensitive to grain-size variation.

**Figure 11:**
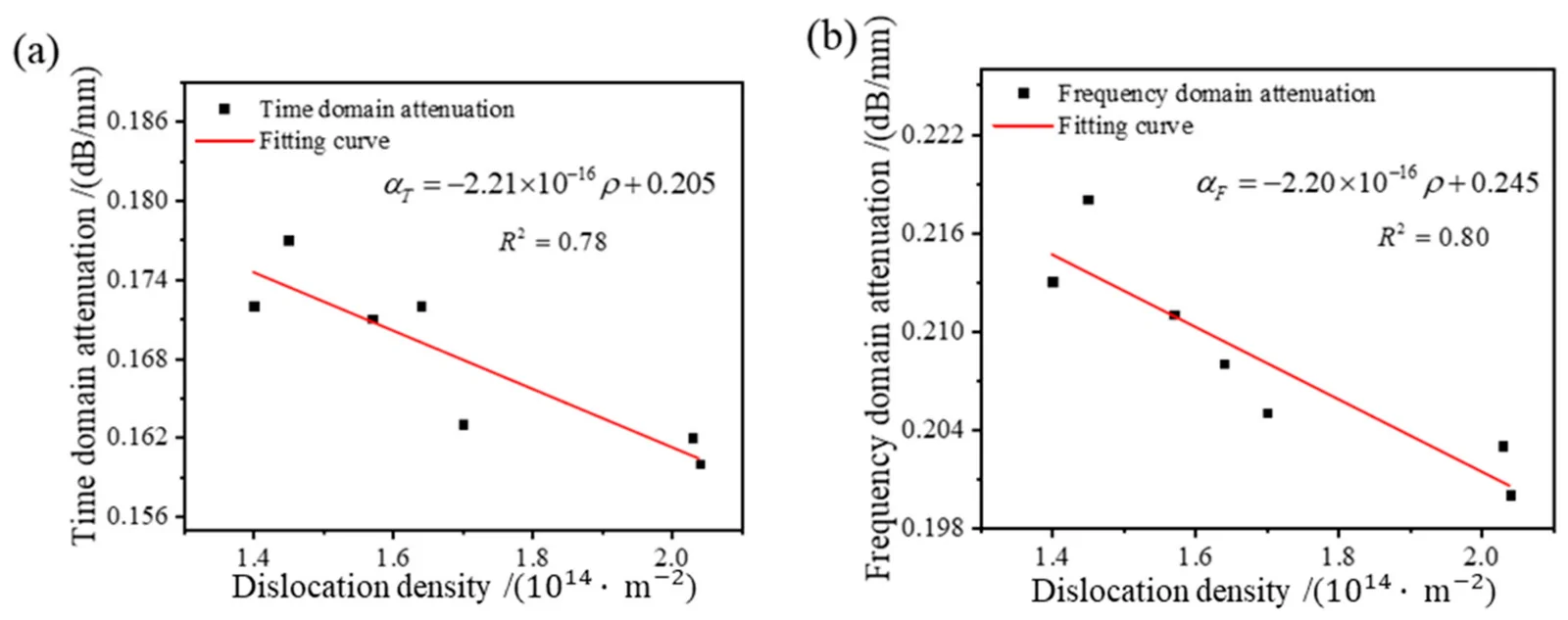
Correlation analysis between the ultrasonic attenuation coefficient and dislocation density: (**a**) time domain attenuation; (**b**) frequency domain attenuation.

To explore the influence of dislocation density on ultrasonic scattering attenuation during the propagation of ultrasonic waves within the material, the correlation between ultrasonic attenuation and dislocation density was analyzed. Espinoza et al. [31] used resonance ultrasound to study the effect of changes in dislocation density in aluminum and copper on ultrasound properties. They found that variations in dislocation density lead to changes in sound velocity. Therefore, to investigate the correlation between ultrasonic attenuation and dislocation density, a linear fitting relationship (9) was employed [32], and the fitting results are shown in Figure 11.

(9) α=dρ+e

where *α* is the ultrasonic attenuation coefficient, *ρ* is dislocation density, and *d* and *e* are constant.

The fitting formulas for the ultrasonic time-domain attenuation coefficient and frequency-domain attenuation coefficient as a function of dislocation density are obtained as follows:

(10) $$\alpha_{T}=-2.21\times 10^{-16}\rho +0.205$$

(11) $$\alpha_{F}=-2.20\times 10^{-16}\rho +0.245$$

The fitting results show that both the ultrasonic time-domain attenuation and frequency-domain attenuation exhibit a negative linear correlation with dislocation density. As the dislocation density increases, both the time-domain and frequency-domain attenuation coefficients decrease. The negative correlation between dislocation density and attenuation coefficient should be understood as an empirical relationship within the present solution-treated sample set. It does not indicate that a decrease in dislocation density alone directly increases ultrasonic attenuation. Instead, dislocation-density reduction occurs simultaneously with grain growth, changes in grain-boundary distribution, and dissolution of cellular substructures during solution treatment [33]. Therefore, the measured attenuation coefficient is governed by the combined microstructural evolution, among which grain-boundary scattering is considered to play a dominant role. The goodness of fit between dislocation density and both ultrasonic time-domain and frequency-domain attenuation is above 0.78.

#### 3.4.2. Laser Ultrasonic Testing of Mechanical Properties

The mechanical properties of PBF-LB 022Cr17Ni12Mo2 steel are governed by multiple strengthening mechanisms, including lattice friction, solid-solution strengthening, cellular-substructure strengthening, dislocation strengthening, grain-boundary strengthening, and possible residual stress effects [34,35,36]. Although the Hall–Petch relationship is commonly used to describe the effect of grain size on yield strength, direct application of the EBSD-equivalent grain size to the Hall–Petch law should be treated with caution in the present PBF-LB material [37]. This is because the grains are elongated and irregular, and the internal cellular substructures and cell-wall dislocation networks may play a more important role in determining yield strength than the apparent EBSD grain size alone.

Therefore, in this study, the relationship between ultrasonic attenuation and yield strength is interpreted as a phenomenological correlation within the present experimental range rather than as a universal Hall–Petch-based prediction model. The decrease in yield strength after solution treatment is mainly associated with dislocation recovery, partial annihilation of cell-wall dislocations, reduction in low-angle grain-boundary fraction, and weakening of cellular substructures. Meanwhile, the ultimate tensile strength remains relatively stable compared with the yield strength, which is consistent with the fact that the characteristic cellular/subgrain scale may not change as markedly as the dislocation density during homogenization treatment. Accordingly, the attenuation-based model established in this work should be regarded as a preliminary calibration relationship for the present solution-treated PBF-LB 022Cr17Ni12Mo2 steel, and further validation using quantitative cell-size measurements, independent sample batches, and residual stress characterization is required.

**Figure 12:**
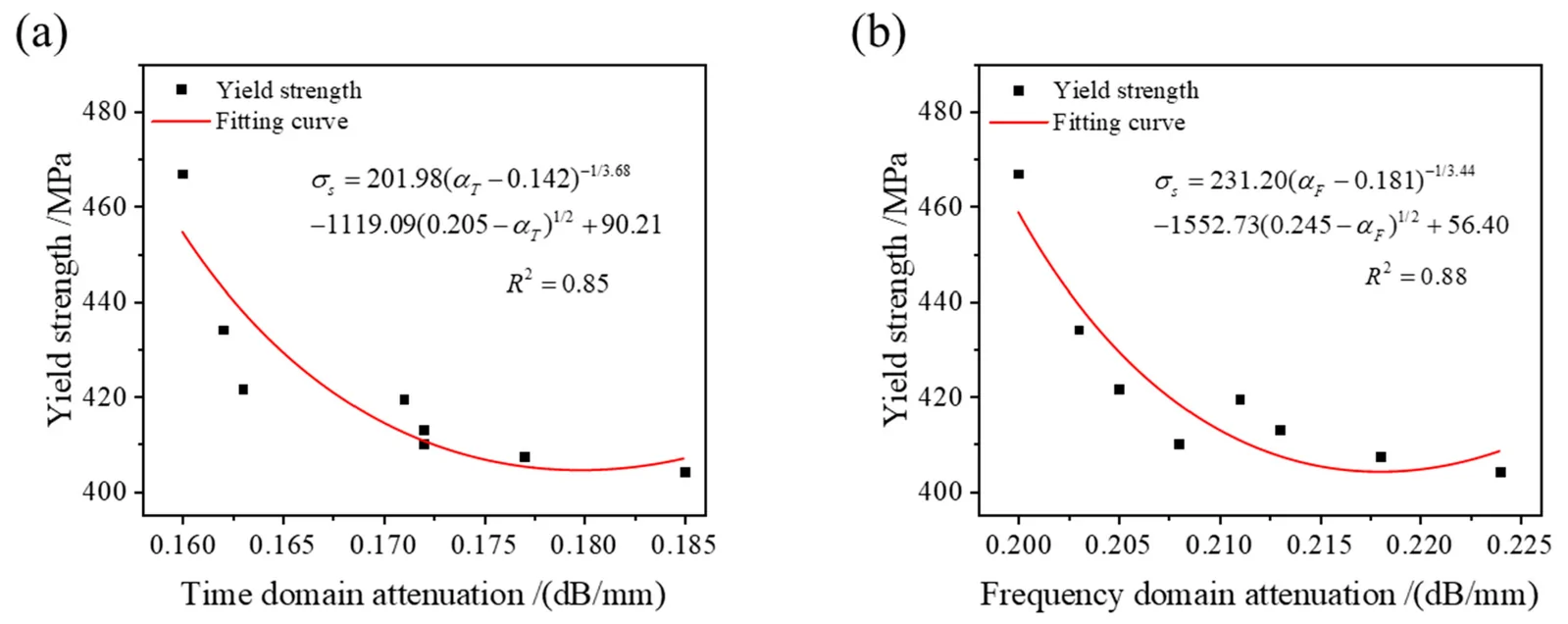
Correlation analysis between yield strength and ultrasonic attenuation: (**a**) time domain attenuation; (**b**) frequency domain attenuation.

Based on the above phenomenological interpretation, the yield strength of PBF-LB 022Cr17Ni12Mo2 steel was empirically fitted using the ultrasonic time-domain and frequency-domain attenuation coefficients, respectively. The fitting results are shown in Figure 12, and the corresponding empirical fitting relationships are given by Equations (12) and (13). The fitting results are shown in Figure 12. The corresponding fitting relationships between yield strength and the ultrasonic time-domain and frequency-domain attenuation coefficients are given by Equations (12) and (13):

(12) $$\sigma_{s}=201.98{\left(\alpha_{T}-0.142\right)}^{-1/3.68}-1119.09{\left(0.205-\alpha_{T}\right)}^{1/2}+90.21$$

(13) $$\sigma_{s}=231.20{\left(\alpha_{F}-0.181\right)}^{-1/3.44}-1552.73{\left(0.245-\alpha_{F}\right)}^{1/2}+56.40$$

The fitting results indicate that the ultrasonic attenuation coefficient can empirically describe the yield-strength variation within the present solution-treated sample set. To assess the in-sample fitting performance of the attenuation-based calibration model, the experimentally measured time-domain and frequency-domain attenuation coefficients were substituted into Equations (12) and (13) to calculate the yield strength within the present dataset. The calculated values were then compared with the experimentally measured yield strengths, and the results are listed in Table 6. It should be noted that this comparison represents an in-sample fitting assessment rather than an independent validation, as the model was established and assessed using the same eight solution-treatment conditions. Therefore, the present model should be interpreted as a preliminary calibration model for describing the yield-strength variation within the current dataset.

As shown in Table 6, the calculated yield strengths obtained from both the time-domain and frequency-domain attenuation models are in reasonable agreement with the experimentally measured values. However, these results should be interpreted as in-sample fitting results rather than independent validation results, because the model was established and assessed using the same eight solution-treatment conditions. The maximum in-sample fitting error was 3.85%, indicating that ultrasonic attenuation can describe the yield-strength variation in the present solution-treated samples [38]. Of the two models, the frequency-domain attenuation model showed slightly better in-sample fitting performance, which is consistent with its higher coefficient of determination. Nevertheless, the present model has not been validated using independent sample batches. Therefore, it should not be directly extrapolated to other PBF-LB processing parameters, heat-treatment conditions, or material batches without further validation.

## 4. Conclusions

In this study, laser ultrasonic testing was combined with SEM, EBSD, XRD, tensile testing, and microhardness measurement to investigate the microstructural evolution, ultrasonic response, and mechanical-property variation in solution-treated PBF-LB 022Cr17Ni12Mo2 steel. Based on the empirical correlations among EBSD-equivalent grain size, KAM-derived dislocation density, ultrasonic attenuation, and yield strength, a preliminary attenuation-based calibration relationship was established within the present set of solution-treated samples. The main conclusions are as follows:

Solution treatment promoted microstructural recovery, weakening of PBF-LB-induced cellular substructures, reduction in low-angle grain-boundary fraction, and partial annihilation of cell-wall dislocations. XRD results showed that the solution-treated samples retained a single-phase γ-austenitic structure, indicating that the changes in mechanical properties and ultrasonic response were mainly caused by microstructural recovery and dislocation-density reduction rather than phase transformation or precipitate dissolution. With increasing solution-treatment temperature, the KAM-derived dislocation density generally decreased from 2.04 × 10^14^ m^−2^ to 1.45 × 10^14^ m^−2^, the yield strength decreased from 466.9 MPa to 407.4 MPa, and the hardness decreased from 246.3 HV to 223.8 HV.

The ultrasonic attenuation coefficient showed empirical correlations with EBSD-equivalent grain size, KAM-derived dislocation density, and yield strength within the present solution-treated sample set. However, the attenuation response should be regarded as a combined effect of grain morphology, cellular substructures, cell-wall dislocation networks, residual pores, texture, and possible LRRS. Therefore, the attenuation-based relationship established in this study is a preliminary, in-sample calibration model rather than a general Hall–Petch-based prediction model. Further studies involving quantitative cell-size measurement, independent validation samples, and residual stress characterization are required.

Overall, laser ultrasonic testing provides a rapid, non-contact, and nondestructive approach with potential for evaluating the microstructure and mechanical-property variation in additively manufactured 022Cr17Ni12Mo2 steel after solution treatment. However, because the present evaluation model was established using a limited dataset of eight heat-treatment conditions, further validation using independent sample batches and larger datasets is required before the model can be applied as a general predictive tool.

## Figures and Tables

**Table 1:** Chemical composition and particle size range of the PBF-LB 022Cr17Ni12Mo2 steel powder.

Element	Cr	Ni	Mo	Fe	Si	Mn	C	P	S
Content (wt.%)	17.00	12.00	2.00	Balance	≤1.00	≤2.00	≤0.03	≤0.045	≤0.03

**Table 2:** Solution treatment process for PBF-LB 022Cr17Ni12Mo2 steel.

	Holding Temperature/°C	Holding Time/Min	Cooling Method
ST1	900	30	Water cooling
ST2	950	30	
ST3	1000	30	
ST4	1050	30	
ST5	1100	30	
ST6	950	60	
ST7	950	90	
ST8	950	120	

**Table 3:** Microstructural parameters of PBF-LB 022Cr17Ni12Mo2 steel after solution treatment.

NO.	Average Grain Size/μm	Low Angle Grain Boundary/%	Dislocation Density /(10^14^·m^−2^)
ST1	12.82	52.0	2.04
ST2	14.51	54.4	1.70
ST3	19.29	52.1	1.61
ST4	14.43	47.6	1.64
ST5	16.31	35.9	1.45
ST6	11.99	59.5	2.03
ST7	15.58	47.5	1.40
ST8	16.22	45.2	1.58

**Table 4:** Mechanical properties of PBF-LB 022Cr17Ni12Mo2 steel after solution treatment.

NO.	Yield Strength/MPa	Ultimate Tensile Strength/MPa	Elongation/%	Hardness/HV
ST1	466.9 ± 1.2	632.3 ± 3.0	51.9 ± 1.3	246.3 ± 5.6
ST2	421.7 ± 3.6	607.1 ± 0.3	62.6 ± 1.1	232.3 ± 6.1
ST3	404.2 ± 2.2	594.0 ± 5.8	68.5 ± 0.3	226.8 ± 3.6
ST4	410.1 ± 3.4	607.2 ± 5.1	66.0 ± 1.4	225.3 ± 4.3
ST5	407.4 ± 8.8	597.9 ± 3.9	64.4 ± 0.4	223.8 ± 3.8
ST6	434.1 ± 0.2	617.2 ± 5.0	55.8 ± 3.0	234.8 ± 7.3
ST7	413.1 ± 1.2	597.8 ± 3.4	69.5 ± 4.9	224.4 ± 5.2
ST8	419.5 ± 1.9	610.0 ± 1.2	59.0 ± 7.2	226.1 ± 4.3

**Table 5:** The characteristic values of the ultrasonic longitudinal wave signal of PBF-LB 022Cr17Ni12Mo2 steel after solution treatment.

NO.	Wave Speed *v*/(m/s)	Time Domain Attenuation		Frequency Domain Attenuation	
		*α_T_* (1P/3P) /(dB/mm)	*α_T_* (3P/5P) /(dB/mm)	*α_F_* (1P/3P) /(dB/mm)	*α_F_* (3P/5P) /(dB/mm)
ST1	5662.95	0.160	0.107	0.201	0.110
ST2	5680.41	0.163	0.075	0.205	0.112
ST3	5696.22	0.185	0.114	0.224	0.161
ST4	5723.01	0.172	0.108	0.208	0.128
ST5	5751.16	0.177	0.096	0.218	0.109
ST6	5665.89	0.162	0.083	0.203	0.108
ST7	5712.88	0.172	0.104	0.213	0.120
ST8	5695.84	0.171	0.087	0.211	0.093

**Table 6:** In-sample fitting results for yield strength calculated using the attenuation-based calibration model.

Sample	Experimental Yield Strength Test/MPa	Calculated Yield Strength by Time-Domain Attenuation Model *α_T_*/MPa	In-Sample Fitting Error/%	Calculated Yield Strength by Frequency-Domain Attenuation Model *α_F_*	In-Sample Fitting Error/%
ST1	466.9	454.6	2.64	458.7	1.75
ST2	421.7	437.9	3.85	429.5	1.86
ST3	404.2	406.9	0.67	408.5	1.06
ST4	410.1	410.7	0.14	418.4	2.02
ST5	407.4	405.2	0.53	404.1	0.81
ST6	434.1	442.9	2.03	439.4	1.22
ST7	413.1	410.7	0.59	407.5	1.36
ST8	419.5	412.5	1.68	410.8	2.07

## Data Availability

The data presented in this study are available from the corresponding author upon reasonable request.
